# The Role of Fecal Microbiota Transplantation in the Treatment of Inflammatory Bowel Disease

**DOI:** 10.3390/jcm10184055

**Published:** 2021-09-08

**Authors:** Magdalena Stojek, Anna Jabłońska, Krystian Adrych

**Affiliations:** Department of Gastroenterology and Hepatology, Medical University of Gdansk, ul. Smoluchowskiego 17, 80-210 Gdańsk, Poland; ajablonska@gumed.edu.pl (A.J.); krystian.adrych@gumed.edu.pl (K.A.)

**Keywords:** fecal microbiota transplantation, inflammatory bowel disease, ulcerative colitis, Crohn’s disease

## Abstract

The exact pathogenesis of inflammatory bowel disease (IBD) is still not completely understood. It is hypothesized that a genetic predisposition leads to an exaggerated immune response to an environmental trigger, leading to uncontrolled inflammation. As there is no known causative treatment, current management strategies for inflammatory bowel disease focus on correcting the excessive immune response to environmental (including microbial) triggers. In recent years, there has been growing interest in new avenues of treatment, including targeting the microbial environment itself. Fecal microbiota transplantation (FMT) is a novel treatment modality showing promising results in early studies. The article discusses the rationale for the use of FMT in inflammatory bowel disease and the yet-unresolved questions surrounding its optimal use in practice.

## 1. Introduction

The exact pathogenesis of inflammatory bowel disease (IBD) is still not completely understood. It is hypothesized that a genetic predisposition leads to an exaggerated immune response to an environmental trigger, leading to uncontrolled inflammation. There are more than 160 known loci associated with IBD, many of which are involved in the gut immune response, including the intestinal barrier, microbial recognition, lymphocyte regulation, and cytokine release [1,2,3]. Increasing incidence of IBD in both industrialized and developing countries suggests the role of some environmental factor, such as changes in diet or the microbial environment [2]. It is not known whether intestinal inflammation results from an abnormal immune response to commensal flora, or whether a primarily imbalanced gut microbiome triggers an aggressive immune response [4].

Current treatment strategies for inflammatory bowel disease focus on correcting the excessive immune response to environmental (including microbial) triggers. In recent years, there has been growing interest in new avenues of treatment, including targeting the microbial environment itself [5]. 

## 2. Human Microbiome

The gut microbiota constitutes the largest population of microorganisms in the human body, with the highest concentration in the colon, where its number reaches up to 10^11^ or 10^12^ cells per gram of colonic content [6]. Molecular analysis of fecal and mucosal samples using 16S ribosomal DNA and RNA showed than the human colon can harbor as many as 36,000 individual species [7]. Firmicutes, Bacteroidetes, Proteobacteria, and Actinobacteria constitute more than 99% of the gut microbiota, with Firmicutes (including Clostridia) accounting for more than 60% of mucosa-attached colonic species, while Enterobacteriaceae such as *Escherichia coli* are a relatively minor subgroup, accounting for only 8% of all bacteria [7,8].

Commensal gut microbiota metabolize nonabsorbable dietary carbohydrates and produce metabolites, including short-chain fatty acids (SCFA) that can account for as much as 10% of the daily energy intake [7]. While SCFAs acetate, propionate, and butyrate are the main energy source for colonocytes, they also enhance mucosal-barrier function by stimulating the secretion of mucus and maintaining the integrity of epithelial tight junctions [9]. Butyrate also binds directly to macrophages and dendritic cells, enhancing the production of anti-inflammatory cytokine IL-10, suppressing proinflammatory cytokine IL-6, and influencing the differentiation and proliferation of regulatory T cells [10,11,12].

Microbiota are also involved in the metabolization of xenobiotic compounds, thus influencing drug metabolism and carcinogenesis [13,14]. Microbial bile salt hydrolases deconjugate primary bile acids and lead to the generation of secondary bile acids that, among other effects, influence the growth of other bacteria [15]. Thus, commensal microorganisms inhabiting the human gastrointestinal tract play an important role in digestion, metabolism, and immune adaptation while also suppressing the growth of competing invasive species [16]. 

In dysbiosis, the decreased concentration of protective SCFA-producing bacteria and the increased accumulation of toxic metabolites of intestinal pathogens lead to mucosal damage, the increased concentration of proinflammatory mediators, and increased mucosal permeability [7,8]. Impaired host immunoregulation with an ineffective downregulation of the innate immune response translates to a loss of tolerance to nonpathogenic microbial antigens and the development of cross-reactive autoimmunity [7]. 

Modern sequencing techniques show that the combined DNA content of the human microbiota (microbiome or metagenome) is significantly more variable than the human genome itself, and it is estimated that only one-third of microbial genes are common to most healthy human hosts [17]. Due to this huge variability in healthy human microbiota, it seems that a “healthy” microbiome cannot be defined by the presence of specific microbial taxa or species, but rather by the ability of the microbial population to perform specific metabolic and other functions while the specific microorganisms performing those functions might differ [17]. It seems evident that a healthy state of the intestine requires a diverse and stable commensal microbial population [17]. 

Decreased microbial diversity of the intestine is found in several diseases of the gastrointestinal tract, including infectious diarrhea, inflammatory bowel disease, and *Clostridioides difficile* (*C. difficile*) infection. Additionally, the intestinal microbial population in many gastrointestinal diseases is characterized by a reduction in the proportion of anaerobic species and an increased proportion of facultative anaerobes such as Proteobacteria and Bacilli [8].

## 3. The Microbiome in Inflammatory Bowel Disease

There is ample evidence for the role of dysbiosis in the pathogenesis of inflammatory bowel disease [7,18]. Several known IBD-susceptibility loci are linked to ways the intestine interacts with the environment [19]. In fact, the first identified CD-susceptibility gene, nucleotide-binding oligomerization domain containing 2 (NOD2), is involved in the immune response to Gram-positive and -negative bacteria [20]. NOD2 mutations influence the abundance of mucosal-adherent bacteria [21] and the transcription of anti-inflammatory cytokine IL-10 [22]. Many studies found that patients with inflammatory bowel disease have fewer SCFA-producing bacteria in their gut, especially *Faecalibacterium prausnitzii*, a symbiotic bacterium with well-documented beneficial effects for the host [23,24].

Some patients with inflammatory bowel disease, including Crohn’s disease and pouchitis, experience clinical improvement with prolonged courses of antibiotics [25,26], while probiotics such as *Escherichia coli* Nissle 1917 have shown promise in the treatment of pouchitis and the maintenance of remission in ulcerative colitis [27,28]. The WHO defines probiotics as “live microorganisms which, when administered in adequate amounts, confer a health benefit on the host” [29]. *Escherichia coli* Nissle 1917 (EcN), named after Professor Alfred Nissle, who first discovered that selected strains of *E. coli* cultured from the stool of healthy humans were able to inhibit the growth of Salmonella and other enteropathogens [30], is one of the most extensively investigated probiotics [31]. The relationship between antibiotic use and IBD is complex. While a recent (within 60 days) exposure to antibiotics was shown to reduce the risk of flares of Crohn’s disease in a case-crossover study [32], a large Canadian case–control study found that patients with a new diagnosis of inflammatory bowel disease were more likely to have been exposed to antibiotics within the preceding 3 to 5 years than patients without IBD, with a dose-dependent relationship between the number of antibiotic dispensations and the risk of IBD diagnosis [33]. 

The inflammatory process in Crohn’s disease, ulcerative colitis, and pouchitis tends to be pronounced in segments of the bowel with the highest concentrations of bacteria [7]. In fact, mucosal biopsies in patients with inflammatory bowel disease show increased concentrations of mucosa-adherent bacteria [21]. The diversion of fecal contents away from an inflamed bowel leads to the healing of inflammation, and inflammation recurs when the mucosa is re-exposed to luminal contents [34]. 

Lastly, patients with inflammatory bowel disease have reduced diversity of the fecal microbiota [35], and differences in microbial diversity were demonstrated even in the same patient between inflamed and noninflamed mucosal samples [36]. The gut microbial flora of patients with IBD are commonly depleted of commensal bacteria such as Firmicutes and Bacteroidetes [37,38]. 

Studies in germ-free mice demonstrated that the presence of gut bacteria is required for the development of IBD-like colitis [39].

## 4. Rationale for Fecal Microbiota Transplantation in Inflammatory Bowel Disease

Current treatment strategies for inflammatory bowel disease focus on the modulation of the patient’s immune response. While the introduction of biologic therapy has significantly improved the overall prognosis in IBD, its use has certain limitations, including considerable rates of primary nonresponse and secondary loss of response [40,41] and despite their overall favorable safety profile, there is concern for adverse effects such as infections and other complications [42]. The ample evidence for the role of dysbiosis in IBD and the disadvantages of available pharmacologic therapies make the prospect of a safe, “natural”, and causative treatment by addressing dysbiosis even more appealing.

## 5. History of Fecal Microbiota Transplantation

The first reports of the therapeutic use of human feces come from China and date back to the 4th century, when so called “yellow soup” (a suspension of human feces) was used to treat food poisoning and diarrhea [43]. Later, Bedouins treated bacterial dysentery with the consumption of fresh camel feces [44]. This remedy was applied by German doctors during World War II, when soldiers were dying of dysentery in Northern Africa. They isolated *Bacillus subtilis* from camel feces used by locals suffering from this disorder and administered it to infected soldiers with good effect [43]. 

The first fecal transfer from healthy to sick animals was reported by the Italian anatomist and surgeon Acquapendente in the 17th century, who called the procedure “transfaunation” [43]. In the 1680s, the Dutch businessman and scientist Antoni von Leeuwenhoek, known for his work in microscopy, discovered microscopic organisms in his own feces [43]. The beginning of the 20th century brought two publications of Russian Nobel Prize winner in physiology, Elie Metchnikoff, who introduced the term “orthobiosis” [45]. His observations of the longevity and health of Bulgarian farmers inspired him to enrich his diet with fermented milk products that improved his general wellbeing [43]. In ”The Prolongation of Life: Optimistic Studies”, he hypothesized that increasing the colonic content of lactic acid bacteria (called *Lactobacillus bulgaricus*) by the consumption of fermented products could postpone aging by protecting patients from toxins released by their native colonic bacteria and promote health [45]. 

Another important observation of the beneficial effects of commensal gut microbiota was made by the German doctor Alfred Nissle during World War I. He isolated an *Escherichia coli* strain (later named “Nissle”) from the stool of a soldier who was the only one who did not suffer from dysentery. Nissle observed that this *E. coli* strain inhibited the growth of *Salmonella enterica* and other bacteria, and conserved this strain in special capsules that he later successfully used for treatment of infectious diarrhea [46]. In 1958, Eiseman et al. reported rapid improvement after the administration of fecal enemas from healthy donors in four critically ill patients with pseudomembranous colitis who had failed to respond to other therapies [44,47].

With the advent of modern antibiotics, FMT remained an obscure form of treatment for many years, until Schwan et al. published the first report of the successful treatment of *C. difficile* infection with FMT via retention enema in 1983 [48]. First-generation FMT, described as “fecal enema”, did not involve any filtering or processing, and there was no standardized donor screening. Over the next several years, FMT was successfully applied via a nasogastric tube [49], gastroscopy, and colonoscopy [50]. Subsequently, FMT oral capsules were shown to be noninferior to delivery by colonoscopy [51]. During this time, the processing and preparation of the donor stool became increasingly involved and sophisticated [52,53].

A recent systematic review and meta-analysis of FMT for the treatment of *C. difficile* infection reported a primary cure rate of 92% across 30 case series and 7 randomized control trials [54]. FMT was demonstrated to be superior to both placebo and vancomycin for recurrent CDI with a number needed to treat (NNT) of 3 [55]. Microbial assays performed in patients undergoing FMT for CDI both before and after the procedure showed a restoration of microbial diversity in recipients to resemble that of donors [56,57].

## 6. Fecal Microbiota Transplantation in Clinical Practice

Because FMT is associated with potential risk of transmission of infectious agents, rigorous donor screening is vital for patient safety. Donors should undergo a thorough clinical evaluation, including a detailed medical history, and blood and stool testing to both prevent the direct transmission of infectious diseases and avoid transferring an adverse microbiota profile that could potentially increase the risk of other diseases associated with abnormal gut microbiota. Diseases of the gastrointestinal tract, autoimmune conditions, neurodegenerative or neurodevelopmental conditions, metabolic disorders, and the use of medications directly affecting gut microbiota disqualify a donor candidate [58]. Donors should be between 18 and 50 years old, as an older age can be associated with adverse changes in the gut microbiota composition [59]. Detailed recommendations for the optimal screening and testing of stool donors and other technical aspects of the FMT procedure were proposed by a recent international consensus conference [58]. 

Since March 2020, the FDA has recommended the additional screening of stool and stool donors for the presence of SARS-CoV-2 infection [60]. In addition to screening donors by the PCR testing of nasopharyngeal swab specimens, assays for the detection of SARS-CoV-2 in human stool specimens are available [61].

The early use of FMT relied on a suspension of fresh stool in saline. Since then, frozen FMT suspensions were demonstrated to have similar efficacy in the treatment of recurrent CDI [62]. For that reason, most FMT procedures for CDI currently utilize donor material from stool banks, which allows for faster access, streamlined, comprehensive testing, and lower overall costs. This also reduces the risk of potential pathogen transmission from the donor to the recipient with fresh FMT by quarantining the frozen stool sample until screening results are available [63]. Occasionally, using a known donor might offer an advantage in special situations such as severe food allergy in the recipient or the treatment of recurrent CDI in an immunosuppressed recipient with no past exposure to EBV/CMV, where selecting an EBV/CMV-negative donor might be preferred [64]. 

The equivalence of fresh and frozen stool samples for FMT in inflammatory bowel disease is less clear. A systematic review and meta-analysis of various protocols of FMT in IBD found different success rates of fresh versus frozen stool FMT in ulcerative colitis (15% pooled clinical remission rate for fresh versus 42% for frozen FMT), while the opposite trend was observed for Crohn’s disease. This finding is difficult to interpret because of potentially confounding variables, such as differing routes and frequencies of administration, and because, for a considerable number of patients with ulcerative colitis (26 out of 225), information about the use of fresh versus frozen FMT was not available [65]. A new systematic review and meta-analysis of studies on FMT in Crohn’s disease also found a significant benefit of fresh compared to frozen stool, with similar caveats [66]. 

FMT can be administered into the upper gastrointestinal tract by upper endoscopy, nasoenteric tube, or the oral ingestion of coated capsules, or into the lower gastrointestinal tract via sigmoidoscopy, colonoscopy, or retention enema. The choice of method partly depends on the clinician’s experience and training [64]. 

Delivery via the upper gastrointestinal tract is available to practitioners who might not be trained in endoscopy, and it was the first method used to demonstrate superiority of FMT over repeated antibiotic administration for recurrent CDI in a randomized controlled trial [67]. The disadvantage of this method is its risk of aspiration, especially with prepyloric application, and the understandable psychological discomfort of the patient who visualizes fecal material being infused into their alimentary tract. This can be prevented by enclosing the FMT material in coated capsules which are then administered orally. The oral administration of coated capsules has the additional advantage of facilitating repeated administration. Special coating techniques that allow for targeted colonic release might permit superior microbial engraftment and reduce the risk of small-intestinal bacterial overgrowth [68]. 

Delivery via the lower gastrointestinal tract can be performed via flexible sigmoidoscopy, colonoscopy, or retention enema. During endoscopy, FMT material is infused directly into the intestinal lumen either in a single portion into the most proximal segment of the colon reached by the endoscope or in several smaller portions throughout the right colon. Administering loperamide allows for slowing the excretion of the donor material, and is hypothesized to increase the chance of successful engraftment [64].

In FMT studies in CDI, the administration of fecal donor material into the lower gastrointestinal tract tended to have higher rates of clinical resolution than those using the upper gastrointestinal route [54,69]. 

The optimal patient preparation is not definitely established. When FMT is used for CDI, antibiotics are usually continued until 1 to 3 days before the procedure to avoid the excessive proliferation of *C. difficile* prior to the engraftment of the donor microbiota. If FMT is administered via colonoscopy bowel lavage, it is thought to both allow for the technical performance of the procedure, and to facilitate engraftment by removing large amounts of the original dysbiotic host flora and CD spores, and washing out residual antibiotics used against CD. Prior to FMT delivered via sigmoidoscopy, purging enemas rather than complete bowel lavage might be sufficient. Bowel lavage is usually not performed before FMT delivered via the upper GI tract [64].

Antibiotic stewardship is important in the immediate postprocedural period. In one study, early antibiotic use within 8 weeks of FMT increased the risk of FMT treatment failure in patients with CDI from 11.3% to 27.6% [70]. When avoiding the use of antibiotics is not possible, narrowing the spectrum of the antibiotics and adjusting the route of administration should be considered [64].

## 7. Safety of Fecal Microbiota Transplantation

Adverse events are usually mild and transient, and are related to the procedure itself. While the general rate of adverse events appears to be higher with the upper GI administration of FMT, serious adverse events are more common with lower GI delivery [71]. 

Common, mild side effects include abdominal cramping, mild diarrhea, bloating, nausea and vomiting, and fever, and are usually self-limiting [71]. Potentially serious side effects include infection, gastrointestinal inflammation (such as appendicitis and diverticulitis), and complications of the procedure itself, including aspiration with upper GI administration and bowel perforation during endoscopy [71]. There is also a potential concern about reactions to antigens in donor stool in patients with a history of severe allergy or anaphylaxis. 

In a systematic review of 50 studies on FMT including 1089 patients, the pooled adverse event rate was calculated as 28.5%. In total, 9.2% of the patients developed serious adverse events (SAEs). Lower GI administration was associated with a 6.1% rate of serious adverse events, while upper GI administration had a 2.0% serious adverse event rate. SAEs attributed to FMT itself included death, secondary infection, IBD flare, autoimmune-disease diagnosis, FMT-related injury, and CDI relapse (0.9%). Out of 38 deaths reported in the study (3.5% total mortality rate), 35 were deemed to be unrelated to FMT and occurred in the course of unrelated conditions, while one occurred due to aspiration during sedation before colonoscopy for FMT, and two deaths due to infection were thought to be possibly related to FMT [71].

Initial data from retrospective studies regarding the outcome of FMT in patients with inflammatory bowel disease treated for CDI showed a significant rate of IBD exacerbations ranging from 18% to 54% [72,73,74]. These high rates might reflect the difficulty in categorizing disease flares with coexisting *C. difficile* colonization, as a subsequent systematic review found a much lower rate of 4.6% of disease flares in high-quality studies and randomized controlled trials (95% CI: 1.8–11) [75]. In a prospective, multicenter cohort study in patients with inflammatory bowel disease treated for recurrent CDI, only 1 out of 34 patients with ulcerative colitis and none of the 15 patients with Crohn’s disease experienced a de novo flare (4% and 0%, respectively), while 62% of ulcerative-colitis patients and 73.3% of Crohn’s disease patients experienced clinical IBD improvement [76]. 

The American Gastroenterological Association has initiated a prospective FMT registry of North American participants receiving FMT for any indication, with the aim of assessing the safety and effectiveness of treatment during an expected follow-up period of 10 years. The registry began enrolling patients in December 2017. According to a 2021 study summarizing the outcomes of 259 patients enrolled in the registry so far, FMT demonstrated a good safety profile. Overall, 45% of participants reported at least one symptom, most commonly diarrhea, abdominal pain, bloating, or constipation. There was a single case of colonoscopic perforation at a biopsy site and two episodes of GI bleeding, namely, one episode of self-limited rectal bleeding and one episode of postpolypectomy bleeding. Infections were reported in 5% of participants during the first month of follow-up, with 1% of infections believed to be related to the procedure [77]. Six patients (4%) had one or more new infections diagnosed between 1 and 6 months. Two participants (1%) were diagnosed with irritable bowel syndrome, and two (1%) had a new diagnosis of ulcerative colitis within 6 months of FMT. While four participants died during follow-up from unrelated conditions, there were no deaths attributed to FMT [77]. 

On the basis of the accumulated data, it appears that FMT is a generally safe procedure, with most adverse effects either mild and transient in nature or directly related to the mode of application. However, the exact role of gut microbiota in the development of many nongastrointestinal diseases is still unknown, and some potential adverse effects might not be apparent in the short term, so caution is maintained, and patients are thoroughly counseled.

## 8. Fecal Microbiota Transplantation (FMT) in the Treatment of Inflammatory Bowel Disease

One of the first instances of FMT for inflammatory bowel disease in modern times was reported by Dr. Justin D. Bennet in 1989 for the treatment of his own intractable, steroid-dependent ulcerative colitis. The treatment proved to be successful, and Dr. Bennet had, at the time of his report, achieved full clinical and endoscopic remission lasting at least 6 months without maintenance medication [78].

In 2003, Borody et al. reported on six patients with ulcerative colitis treated with repeated retention enemas of donor fecal material who experienced clinical improvement within a week and complete resolution of symptoms within four months. The longest follow-up in this study was 13 years, with the patient in full remission for all of this time [79].

Over time, several noncontrolled case series and cohort studies showed promising results, with a 2014 meta-analysis reporting a pooled estimate for achieving clinical remission after FMT of 36.2% for inflammatory bowel disease, and 22% for ulcerative colitis in cohort studies [80]. 

Additional data on FMT in patients with IBD came from studies in patients treated for CDI. FMT was less effective for the prevention of CDI recurrence in patients with IBD, and between 18% and 54% of patients experienced IBD flares [72,73,74]. The increased rate of IBD flares might have been overestimated due to using PCR testing as evidence for CDI infection, so that some of the patients might have had an IBD flare rather than CDI from the beginning. A later review of RCTs and high-quality studies showed a much lower rate of IBD exacerbation after FMT [75].

Then, 2015 saw the publication of the first two randomized controlled trials on the use of FMT in ulcerative colitis [81,82]. 

Moayyedi et al. [81] enrolled 75 patients with active ulcerative colitis. The treatment group (38 patients) received FMT from healthy anonymous donors via rectal enema once weekly for 6 weeks, while patients in the placebo group (37 patients) were given water enemas in the same intervals. Response to treatment was evaluated by sigmoidoscopy at 7 weeks. There was no difference in serious adverse events between the FMT and placebo groups. Of patients in the treatment group, 24% compared to 5% in the placebo group achieved complete remission, defined as a total Mayo score of 3 points and an endoscopic subscore of 0 points, i.e., endoscopic remission. Patients on immunosuppressive therapy and patients with a more recent diagnosis of UC were more likely to respond to FMT. Out of nine patients who achieved remission with FMT, seven had complete histologic remission, meaning no active inflammation in any of the biopsies, and two had mild microscopic-only inflammation. Eight of the nine patients remained in remission at week 52, with four of them discontinuing all their medications for ulcerative colitis. Interestingly, seven out of nine patients who achieved remission with FMT had received fecal material from the same donor. The successful donor had a treatment success rate of 39% (7 out of 18 patients), while the remaining donors had a pooled success rate of 10% (2 out of 20 patients). 

Donor-stool analysis found significant differences in the taxonomic profiles between the most successful donor (whose stool was rich in Lachnospiraceae and Ruminococci) and another, less successful donor who provided most of the remaining donations, and whose stool was enriched in Escherichia and Streptococci. The study was terminated early for futility, while patients who were already enrolled were allowed to complete their treatment, with a subsequent increase in overall success rate. Most of the successful treatments were completed after the enrollment had already stopped. Even though the remission rate in this study was relatively modest, it was partly due to the stringent definition of treatment success, i.e., full endoscopic remission, and a relatively small enrollment. Additionally, more patients in the FMT group compared to the placebo group had extensive colitis (62.5% vs. 37.5%). The authors speculated that the efficacy of FMT may be donor-dependent, and this may explain why some case series have shown promise, and others have had disappointing results.

Published in the same issue of *Gastroenterology* in 2015, the study by Rossen et al. [82] enrolled 50 patients with mild to moderately active ulcerative colitis, 48 of whom completed the study. The treatment group (23 patients) received FMT from healthy donors via nasoduodenal tube at the start of the study 3 weeks later; in the placebo group (25 patients), FMT was performed with autologous fecal microbiota. Response to treatment was evaluated by sigmoidoscopy at week 12. The composite primary end point was clinical remission (simple clinical colitis activity index scores ≤ 2) combined with a ≥ 1 point decrease in the Mayo endoscopic score at week 12. Serious adverse events occurred in four patients (two in the FMT group), but these were not considered to be related to the FMT. Clinical remission was achieved in 7 out of 23 patients who had received donor FMT (30.4%), and 5 out of 20 patients who had received autologous FMT (20%), and no statistically significant differences were demonstrated regarding clinical or endoscopic remission. Patients in the donor FMT group who did achieve remission had undergone a shift in their microbial profile towards that of their healthy donors; remission was associated with proportions of Clostridium clusters IV and XIVa, and the microbiota of responders was different from that of nonresponders. As the study used 15 different donors, it was not possible to determine the presence of a superdonor effect due to a low number of procedures per donor. 

In 2017, Paramsothy et al. [83] reported the results of a large multicenter trial where 41 patients received FMT via one-time colonoscopic infusion followed by retention enemas 5 times per week for 8 weeks, while 40 patients were randomized to receive placebo infusion and enemas. Endoscopic response was assessed at week 8. Of 41 patients treated with donor FMT, 11 (27%) achieved steroid-free clinical and endoscopic remission compared to 3 out of 40 patients in the placebo group (8%). Both groups had similar rates of adverse events (78%, FMT; 83%, placebo), which were mild and self-limiting in the vast majority of cases. Serious adverse events were observed in two patients in the treatment group compared to one patient in the placebo group. Stool analysis showed increased microbial diversity with fecal microbial transplantation. Patients in this study received pooled FMT material from up to seven different donors. Despite using multiple donors, and although the pooled stool mixture had increased microbial diversity compared to individual stool samples, clinical and endoscopic remission rates were similar to those obtained in earlier studies that used single donors. While the overall efficacy was not improved with using multiple donors, the study demonstrated a varied response rate depending on the composition of FMT donor material, as different stool batches produced different effects. A superdonor effect was identified, with patients who had received FMT batches containing the stool from the superdonor achieving a 37% remission rate compared to 18% in those whose FMT batches did not include stool from the superdonor [83,84]. 

In 2019, Costello et al. [85] published the results of a multicenter study involving 73 patients with mild to moderately active ulcerative colitis, who were randomized to receive anaerobically prepared pooled donor FMT (38 patients) or autologous FMT (35 patients) administered via colonoscopy, followed by two enemas over 7 days. Open-label donor FMT was offered to patients in the autologous FMT arm at 8 weeks. The patients were followed for 12 months. The primary outcome was steroid-free remission at 8 weeks, defined as a total Mayo score of no more than 2 points and an endoscopic Mayo subscore of 0 or 1. Remission was achieved in 12 out of 38 donor FMT patients (32%), with five still in remission after 12 months (13%) compared to only 3 of the 35 patients (9%) receiving autologous FMT. There were 3 serious adverse events in the donor FMT group and 2 in the autologous FMT group.

While the initial clinical and endoscopic remission rates in high-quality studies on FMT in ulcerative colitis seem promising, it is important to determine the duration and stability of its effect. In 2019, Sood et al. published the results of a pilot study for the use of FMT in maintenance of remission in patients with previously successful induction FMT [86]. The aim of the study was to assess the potential of FMT in maintaining steroid-free remission in ulcerative colitis. The study enrolled 61 patients with UC in clinical remission, achieved after a repeated application of FMT, who were randomized to maintenance FMT or placebo colonoscopic infusion every 8 weeks for 48 weeks. The study examined the rates of clinical (total Mayo score no more than 2, with all subscores ≤ 1), endoscopic, and histologic remission at week 48. Out of 31 patients randomized to receive FMT, 27 (87.1%) achieved clinical remission compared to 66% (20 out of 30) of patients assigned placebo. Endoscopic remission was achieved in 58.1% (vs. 26.7% with placebo) and histological remission in 45.2% (vs. 16.7% with placebo). An exacerbation of disease occurred in three patients on maintenance of FMT (9.7%) and eight patients on placebo (26.7%). The study demonstrated the efficacy and safety of repeated FMT in maintaining remission in ulcerative colitis, and achieving endoscopic and histological remission. 

Lastly, a recent study by Brezina et al. [87] examined the efficacy and safety of FMT enemas compared to topical 5-ASA in patients with left-sided ulcerative colitis (FACTU, fecal bacteriotherapy for ulcerative colitis). In this open-label randomized noninferiority trial, patients with clinically and endoscopically active left-sided ulcerative colitis (with a total Mayo score between 4 and 10 and endoscopy subscore of no less than 2) were randomized to either FMT enemas (five in the first week and once weekly in the following 5 weeks, 10 infusions total) or 4 g mesalamine enemas daily for 2 weeks and then every other day until the end of week 6. Enema tolerance was defined as retaining the enema for at least 15 min. Endoscopic disease activity was assessed by sigmoidoscopy at weeks 6 and 12, and the total Mayo score was calculated. Clinical remission was defined as a total Mayo score of no more than 2 with no subscore above 1 at week 12, and clinical response as a reduction in total Mayo score of at least 2 points. Endoscopic remission was defined as an endoscopic Mayo score of 0 at both weeks 6 and 12.

Healthy donors were identified and selected locally by each center. After rigorous screening, stool samples from prospective donors were analyzed by 16S rRNA sequencing, and the donor with the greatest microbiome diversity was selected, along with an alternate. Those donors were then used for all the patients in a given center, with each patient receiving FMT from the same donor over the entire study. After preparation, the infusions were frozen and thawed immediately before the procedure.

The study had enrolled 45 patients who were randomly assigned to receive either FMT (*n* = 23) or 5-ASA enema (*n* = 22). Two patients in the FMT group did not tolerate the first enema and were not included in the final analysis. Of the FMT patients, 57% (12 out of 21) and 36% of the patients receiving topical 5-ASA (8 out of 22) achieved clinical remission at week 12. The noninferiority of FMT with 10% margin was confirmed (95% CI: −7.6%, 48.9%). Complete endoscopic remission was achieved at week 6 by three FMT patients (14%) and one 5-ASA patient (5%), *p* = 0.34), and at week 12 by three FMT patients (14%) and three 5-ASA patients (14%, *p* = 1.0).

The only serious adverse event observed during the study was the worsening of colitis necessitating treatment escalation, which occurred in four patients receiving FMT and one patient on 5-ASA enemas. Microbial assays were performed by high-throughput sequencing in 35 of the study participants before and after treatment. The FMT responders showed decreased abundance of order Bacteroidales and family *Bacteroidaceae,* and increased microbial diversity after treatment. No significant changes in the microbial taxa were seen after topical 5-ASA treatment. 

An interesting aspect of the study is that it compared FMT to active treatment. While it was not sufficiently powered to detect the superiority of FMT over topical 5-ASA, the high rate of treatment success is encouraging, and the authors are planning a larger study in this indication.

While most trials of FMT in IBD involve patients with ulcerative colitis, there are fewer data on its role in Crohn’s disease. The variable disease location makes it difficult to compare individual patients, and can influence the results depending on the route of FMT delivery, although a recent study by Yang et al. showed no significant differences in clinical remission rate and adverse events in 27 patients who had received FMT via gastroscopy or colonoscopy [88]. Planning good-quality trials with comparable patient groups seems extremely difficult in that setting. Proving endoscopic remission can also be much more challenging in CD than in UC. Published reports show heterogeneous results, with some cases of clinical deterioration after FMT in CD [89,90].

In 2017, Paramsothy et al. [90] published a systematic review and meta-analysis assessing the effectiveness and safety of FMT in various IBD subtypes. They included 53 studies (41 in UC, 11 in CD, 4 in pouchitis) that substantially varied in methodology. Clinical remission was achieved in 36% (201/555) of UC, 50.5% (42/83) of CD, and 21.5% (5/23) of pouchitis patients. Subgroup analyses in the UC population suggested a positive correlation of clinical remission with increased number of FMT infusions and lower gastrointestinal tract administration. The authors warned that the reported clinical remission rates in the CD population should be interpreted with caution, considering the wide confidence intervals and the presence of publication bias. Clinical remission does not always correlate with endoscopic remission, especially in CD. Follow-up endoscopy was performed in all four RCTs and 6 of the 24 cohort studies of FMT in UC. Endoscopic outcomes were better in the FMT group that had received multiple lower gastrointestinal FMT infusions. Only one CD study presented endoscopic outcomes, and none of the six examined patients showed endoscopic remission. The limited number of studies on pouchitis precluded a meta-analysis. The majority of studies did not report FMT-related serious adverse events (one death due to toxic megacolon and sepsis, one patient with aspiration pneumonia after nasogastric FMT infusion, and a few cases of disease worsening).

The largest published study on FMT in CD refractory to standard treatment included 30 patients [91]. In this group, a single standardized FMT from a single donor was performed via the midgut by a tube during gastroscopy. The highest clinical improvement (86.7%) and clinical remission (76.7%) were observed within the first month after FMT and gradually decreased during a 6-to-15-month follow-up (66.7% and 60%, respectively, after 6 months). FMT was notably effective in relieving CD-related abdominal pain. The study also found an improvement in body weight, hemoglobin, albumin, and lipid profile in a 3-month observation. There were no severe adverse events during the FMT procedure and in a 6–15 month follow-up. Fresh stool FMT was more effective than frozen stool FMT, though the difference was not statistically significant.

The only randomized single-blind sham-controlled trial evaluating the role of FMT in CD published so far had a completely different design [92]. A single FMT or a sham transplant was administered via colonoscopy in patients with colonic or ileocolonic CD who had achieved clinical remission with systemic corticosteroids before the procedure. The authors assumed that performing FMT in patients who had achieved remission might be more effective in sustaining remission and safer than during an active flare of the disease. The primary endpoint was the donor microbiota engraftment at week 6, but none of the patients achieved that outcome. The steroid-free clinical remission rate at 10 and 24 weeks was 44.4% and 33.3%, respectively, in the sham transplantation group, and 87.5% and 50.0%, respectively, in the FMT group, but this difference was not statistically significant. The authors suggested that this lack of statistical significance might have resulted from the small number of patients enrolled in the study (eight patients in the FMT group and nine patients in the sham group). On the other hand, there was a significant benefit of FMT over sham with respect to endoscopic disease activity and CRP levels, suggesting a better control of inflammation in the FMT group. There was a positive correlation between higher colonization by donor microbiota and maintenance of remission. Two of the patients were not colonized by the donor’s microbiota at all, and they experienced an early CD flare, similar to patients in the sham transplantation group. No significant differences were found in the microbial profile between effective and ineffective FMT donors, while higher baseline levels of several taxa belonging to the Gammaproteobacteria class of the Proteobacteria phylum in the recipients were predictive of the nonengraftment of donor microbiota. 

The authors speculated that the low similarity index between donor and recipient microbiota may have been the reason for the insufficient effect of a single infusion, and that repeated FMT infusions should be evaluated in this setting.

Recently, a new systematic review and meta-analysis was published evaluating the efficacy and safety of FMT in CD patients [66]. Because most published studies lack control groups, contain small numbers of patients, and use various FMT protocols, the quality and amount of available data are limited. Lastly, only 12 trials were included in the analysis (one RCT, seven cohort studies, and four case studies). The overall clinical remission and clinical response rate of CD patients was 0.62, and 0.79, respectively. Remission was related to a positive change in the gut microbiome. 

In most studies, patients received a single infusion of FMT. The authors suggested that repeat FMT infusions might be necessary to maintain a long-term clinical response as in some UC studies. Fresh stool FMT was associated with higher clinical remission than that in frozen stool FMT (73% vs. 43%; *p* < 0.05). Most of the adverse events were mild and self-limiting. A short follow-up, small number of patients, and the lack of a control arm in most studies impede assessment of FMT-related adverse events.

Another potential indication for FMT in IBD is pouchitis. It occurs in up to 60% of UC patients after restorative proctocolectomy and is the most common long-term complication in this group [93]. The exact pathogenesis of pouchitis remains unclear, but dysbiosis seems to be a key factor. This hypothesis is supported by differences in the microbiota composition between patients with pouchitis and those with a noninflamed pouch, and the role of antibiotics in the treatment of pouchitis and probiotics in the prevention of relapses [94,95]. A recent systematic review evaluating the role of FMT in the treatment of chronic pouchitis included only nine studies eligible for the review, so reliable data in this indication are limited [93]. Generally, clinical response to FMT was reported in 14 (31.8%) out of 44 patients at different time points after FMT, and clinical remission in 10 (22.7%) patients. Reported adverse events were minor and self-recovering. Only one randomized controlled trial was available for the review, but it showed no beneficial effect of FMT and was prematurely terminated [96]. The authors suggested that such poor therapeutic effect resulted from exceptionally low donor microbial engraftment in the ileal pouch (only one out of six patients successfully engrafted FMT). Well-designed controlled trials are needed to evaluate the real effect of FMT in patients with pouchitis and the optimal therapeutic strategy, such as the route of FMT delivery, the number of procedures, and donor selection.

A summary of randomized controlled trials in FMT for inflammatory bowel disease is shown in Table 1.

### Optimizing FMT Strategy and Donor Selection

The optimal FMT protocol for IBD that would maximize the donor microbiome engraftment remains uncertain. Recently, a systematic review and meta-analysis was published to evaluate the effects of antibiotic pretreatment and repeated FMT dosing in IBD treatment. Mocanu et al. [97] compared relapse and remission rates in patients after a single FMT versus repeated FMT infusions and antibiotic pretreatment. They reviewed 28 studies containing 976 patients, 22 studies including patients with UC, four with CD, and two with both UC and CD. Of the patients, 41.9% (*n* = 409) were treated with a single FMT, and 30.0% (*n* = 229) with repeated FMT infusions. Pooled response and remission rates were greater for patients receiving repeated FMT (70% and 43%, respectively) compared to a single FMT administration (53% and 30%, respectively). This difference was even more favorable when the subgroup of patients with UC was analyzed. Only 11.2% (*n* = 109) of patients received antibiotic treatment prior to FMT. Pooled response and remission rates were greater for patients after antibiotic pretreatment (82% vs. 58% and 66% vs. 31%, respectively). The duration of antibiotic pretreatment and types of used antibiotics were variable. Reported adverse evets were generally mild and self-limiting (i.e., transient fever or gastrointestinal symptoms). Serious adverse events were reported in 26 studies, but none was deemed related to FMT (13 patients underwent colectomy for UC, one was diagnosed with *Clostridioides difficile* infection, and one with cytomegalovirus infection). Mocanu et al. concluded that, although their results seem promising, they must be interpreted with caution in the context of the heterogeneity of available studies, and highlighted the need for the standardization of future FMT protocols. More studies are necessary to indicate whether the benefit of antibiotic pretreatment outweighs the potential complications associated with the induction of antibiotic resistance or CDI.

Overall, the clinical response rate for FMT for inflammatory bowel disease is modest, and there is a high variability of patient responses, even among patients undergoing the same treatment protocol [84]. Unlike CDI, where FMT is associated with a high success rate after a single procedure [54,98], and the microbial diversity is quickly restored [99], therapeutic success of FMT is harder to predict. In particular, several studies identified significant variations in clinical response between different donors [81,83]. The term “superdonor” was proposed to describe donors whose stool contributed to greater rates of successful FMT outcomes [84]. Effective donors have significantly higher bacterial richness and diversity, and successful transplants are associated with a higher number of transferred bacterial phylotypes [100]. FMT recipients who experience clinical improvement (responders) undergo a greater increase in gut-microbiota diversity compared to nonresponders [100,101]. Specific bacterial genera within *Clostridium* clusters *IV* and *XIVa* were identified in both effective donors and successful recipients [81,82,83,100,102]. Even so, it is not currently possible to reliably predict the effectiveness of a given donor before FMT for IBD. 

In the 2017 study by Paramsothy et al., where material from several stool donors was pooled to increase the chance of successful engraftment [83], post-FMT remission rates (27% for FMT versus 8% for placebo) were similar to those of previous studies that used stool from a single donor. It seems, therefore, that a favorable shift in the microbial environment is not a matter of simple enrichment of missing strains. Engraftment may be partially dependent on the microbial interactions between donor and recipient strains. In other words, strain incompatibilities between FMT donor and recipient may impede microbiome engraftment [103]. The host immune response to the transplanted microbiota may lead to FMT rejection. In a small 2017 pilot study, Ponce-Alonso et al. isolated lymphoid cells from the rectal biopsies of a patient with ulcerative colitis, and separately incubated them with gut microbiota samples from three potential stool donors. Interleukin assays in supernatants were used to determine the most compatible FMT donor. FMT was then successfully performed, resulting in optimal engraftment and good clinical response [104]. A follow-up study of immunologic FMT compatibility testing involving four patients with ulcerative colitis showed no clinical improvement in any of the patients, underscoring the need for further research in this area [105].

Other host-related factors may also play a role, in that different patients might lack different microbial taxa and thus respond to different donors. The efficacy of FMT might depend on the restoration of missing metabolic deficits rather than specific strains, and the optimal treatment of chronic diseases associated with microbial dysbiosis may involve matching the patient to a specific donor on the basis of the metabolic deficiency that needs to be corrected (dysbiosis-matched FMT) [84].

Another important aspect of FMT for inflammatory bowel disease is the difficulty of achieving sustained remission. The successful engraftment of FMT in this context requires repeated dosing over a prolonged period of time, and the duration of response is variable. In an observational study by Li et al., 202 patients were followed after an FMT procedure for ulcerative colitis, with a second FMT treatment offered in case of relapse. The median time of sustained clinical response, defined as a decrease in partial Mayo score of 3 or more points and at least 20% from baseline, with a rectal-bleeding subscore of 0 or 1, and a decrease in the rectal-bleeding subscore by at least 1 after the first FMT procedure was 120 days; the median time of sustained clinical response after the second procedure was 182.5 days [106].

Similarly, follow-up data from the 2017 FOCUS study by Paramsothy et al. revealed that the median time to disease relapse (defined as worsening symptoms requiring the escalation of treatment) among 35 patients who had originally achieved steroid-free clinical remission after FMT was 6 months. Remission for longer than 12 months was observed in 12 patients (15.4% of FMT recipients), three of whom had self-initiated further FMT during that time. Three patients (3.8% of FMT recipients) had sustained clinical remission over 5 years [107]. Of the 78 patients who had originally received FMT (68 of whom were available for follow-up), 29 were on biological medications at the time of follow-up, six underwent a proctocolectomy for disease worsening, and one additional patient had surgery for colonic adenocarcinoma. There was also a new diagnosis of fibromyalgia deemed possibly related to FMT, and a case of a perianal fistula attributed to chronic trauma from self-administered enemas. The authors concluded that the response to fecal microbiota transplantation in ulcerative colitis is not sustained in the long term following induction therapy.

## 9. Future Perspectives

The role of FMT in the treatment of inflammatory bowel disease is still evolving. While the modification of adverse gut microbiota remains an important area of research and a promising treatment strategy, the optimal management of dysbiosis in IBD is still unknown. In view of the low remission rates and variable treatment response, FMT is currently not a treatment option for inflammatory bowel disease, and can only be offered to patients as part of a clinical trial. There is currently less high-quality evidence for the benefit of FMT for Crohn’s disease compared to ulcerative colitis, especially with regard to endoscopic response, and the small study sizes and heterogeneity of disease presentations make it challenging to demonstrate such a benefit at present. Many unresolved questions about various aspects of FMT remain to be settled, including the identification of FMT-responsive patients, optimizing donor selection, choosing the most effective dose, the type of stool preparation, FMT administration route, pretreatment, and timing, and understanding both short- and long-term safety [108]. The most likely scenario is that FMT remains a temporary form of therapy, and will be replaced by more targeted and specific microbial products [109]. In the meantime, the methodology needs to be optimized and standardized, so that patients receive the best possible treatment. More high-quality studies are needed to specify the optimal interventions in selected groups of patients depending on the indication for FMT. There are still insufficient data on the long-term efficacy and safety of FMT in IBD, pending results of long-term follow-up registries. The COVID-19 pandemic and the detection of SARS-CoV-2 genetic material including live virus in stool even after the resolution of respiratory symptoms have raised concerns for the potential transmission of yet-unknown future pathogens [110].

## Figures and Tables

**Table 1:** Characteristics of published randomized controlled trials on fecal microbiota transplantation (FMT) for inflammatory bowel disease (IBD): ulcerative colitis (UC), Crohn’s disease (CD), and pouchitis.

Disease	Study	No. of Patients (FMT/Control)	Disease Activity	Delivery Route	Frequency	Donor	Follow-Up	Response (FMT/Control)	Remission (FMT/control)	SAEs (FMT/Control)
UC	Moayyedi et al., 2015 [81]	75 (38/37)	Mild to moderate	Retention enemas	6 weekly	Single	7 weeks	39%/24%	24%/20%	3/2
UC	Rossen et al., 2015 [82]	48 (23/25)	Mild to moderate	Nasoduodenal infusions	Twice—at week 0 and 3	Single	12 weeks	47.8%/52%	30.4%/20%	2/2
UC	Paramsothy et al., 2017 [83]	81 (41/40)	Mild to moderate	Colonoscopy followed by enemas	5 enemas per week for 8 weeks (40)	Pooled multidonor	8 weeks	54%/23%	27%/8%	2/1
UC	Costello et al., 2019 [85]	73 (38/35)	Mild to moderate	Colonoscopy followed by enemas	2 enemas over 7 days	Pooled multidonor	8 weeks	55%/20%	32%/9%	3/2
UC	Sood et al., 2019 [86]	61 (31/30)	Remission	Colonoscopic infusions	Every 8 weeks for 48 weeks	Single	48 weeks	-	87.1%/66.7%	0/0
UC	Brezina et al., 2021 [87]	43 (21/22)	Mild to moderate left-sided	Retention enemas	5 times in the first week then once weekly for 5 weeks	Single	12 weeks	71%/55%	57%/36% (noninferiority of FMT with 10% margin)	4/1
CD (colonic/ileocolonic)	Sokol et al., 2020 [92]	17 (8/9)	Remission	Colonoscopic infusion	Once	Single	24 weeks	-	50%/33.3%	0/0
Pouchitis	Herfarth et al., 2019 [96]	6 (4/2)	Antibiotic-dependent pouchitis	Single endoscopic infusion followed by capsules	6 capsules daily for 2 weeks	Single	21 days (prematurely terminated)	0/0 (1/6 patients after repeated FMT in open label extension)	0/0 (1/6 patients after repeated FMT in open label extension)	0/0

## Data Availability

Data sharing is not applicable to this article.
